# Special needs dentistry training and education for preparedness in the oral health therapy curriculum

**DOI:** 10.3389/froh.2026.1896214

**Published:** 2026-09-24

**Authors:** Dorothy Koh, Delyse Leadbeatter, Melanie Aley, Woosung Sohn, Archana Pradhan

**Affiliations:** Faculty of Medicine and Health, School of Dentistry, The University of Sydney, Sydney, NSW, Australia

**Keywords:** curriculum, oral health therapy, people with disability, preparedness, special needs dentistry

## Abstract

**Background:**

Individuals with disability experience disproportionately poorer oral health outcomes and face significant barriers in accessing dental care. Despite increasing recognition of Special Needs Dentistry (SND) as a priority area, gaps remain in the education and preparedness of the oral health workforce, particularly oral health therapists (OHTs). This article addressed the need for structured, evidence-informed curriculum models to support the integration of SND into oral health education.

**Aim:**

Guide the integration of a consensus-based SND curriculum framework into oral health therapy programs.

**Methods:**

A multi-phase research approach was undertaken, including a scoping review, curriculum mapping and content analysis of oral health programs, workforce and student perception surveys, and a Delphi study with expert stakeholders. Findings across phases were synthesised to inform the development of the curriculum framework.

**Results:**

Consistent gaps were identified across all phases, including variability in SND curriculum content, limited clinical exposure, and low levels of perceived preparedness among students and practitioners. Strong interest in further SND training was observed. The resulting framework comprises ten competency domains encompassing communication, behavioural management, clinical care, interprofessional collaboration, and ethical practice. Learning outcomes are structured across undergraduate and postgraduate levels and aligned with established educational principles, including scaffolded learning and competency-based progression. Suggested learning activities and assessment tasks are provided to support implementation.

**Conclusion:**

The proposed SND curriculum framework offers a practical and adaptable model for integrating disability-inclusive education into oral health programs. By supporting the development of knowledge, clinical competence, and professional confidence, the framework has the potential to strengthen workforce capability, reduce reliance on specialist services, and improve access to equitable oral healthcare for people with disability. Further research is required to evaluate its implementation and impact in educational settings.

## 1 Introduction

The oral health needs of individuals with disability and additional healthcare needs represent a growing and often underserved area within contemporary healthcare systems (1). This population experiences disproportionately higher rates of oral disease, alongside significant barriers to accessing appropriate dental care (2). These challenges are multifactorial, encompassing physical, behavioural, and systemic factors, and are further compounded by a workforce that frequently reports limited preparedness and confidence in managing patients with disability (3).

Although Special Needs Dentistry (SND) has gained recognition as a specialty in recent years, access to practitioners with advanced training in this field remains limited. In Australia, there are currently only 29 registered specialists in SND, highlighting the need to expand workforce capacity beyond specialist services (4). Accordingly, strengthening education in SND is essential not only for dentists but also for the broader oral health workforce, particularly oral health therapists (OHTs). OHTs are well-positioned to contribute to preventive care, oral health promotion, and continuity of care through collaboration with carers, support workers, and the healthcare team. With appropriate training and clinical exposure, they have the potential to support more inclusive and accessible models of care (5).

Despite increasing awareness of oral health disparities, significant gaps persist in the education and training of the oral health workforce, particularly in preparing graduates to manage patients with disability in clinical practice (6). There is a lack of structured, evidence-informed curriculum models to guide the integration of SND into oral health training. Existing educational approaches are often variable in both content and depth, limiting consistency in competency development across programs (7). To address these gaps, a multi-phase research program was undertaken to investigate the educational preparation, perceptions, and potential role of the oral health workforce in SND, and to inform the development of a comprehensive curriculum framework for oral health therapy programs (8). The research incorporated a scoping review (9), curriculum mapping and content analysis (10), workforce and student perception studies, and expert consensus through a Delphi process (11). Across these phases, consistent findings emerged, including variability in SND curriculum content, limited clinical exposure, and low levels of perceived preparedness among students and practitioners. Importantly, strong interest in further training suggested that willingness to provide care is not the primary barrier; rather, the absence of structured and clinically grounded education constrains workforce engagement in SND. Each phase of the research program informed specific components of the curriculum framework. Collectively, these phases were designed to move from understanding the problem to identifying educational gaps and to informing a practical educational response (Figure 1). The research culminated in the development of a consensus-based curriculum framework designed to support the progressive development of knowledge, clinical skills, and professional competencies in SND.

**Figure 1:**
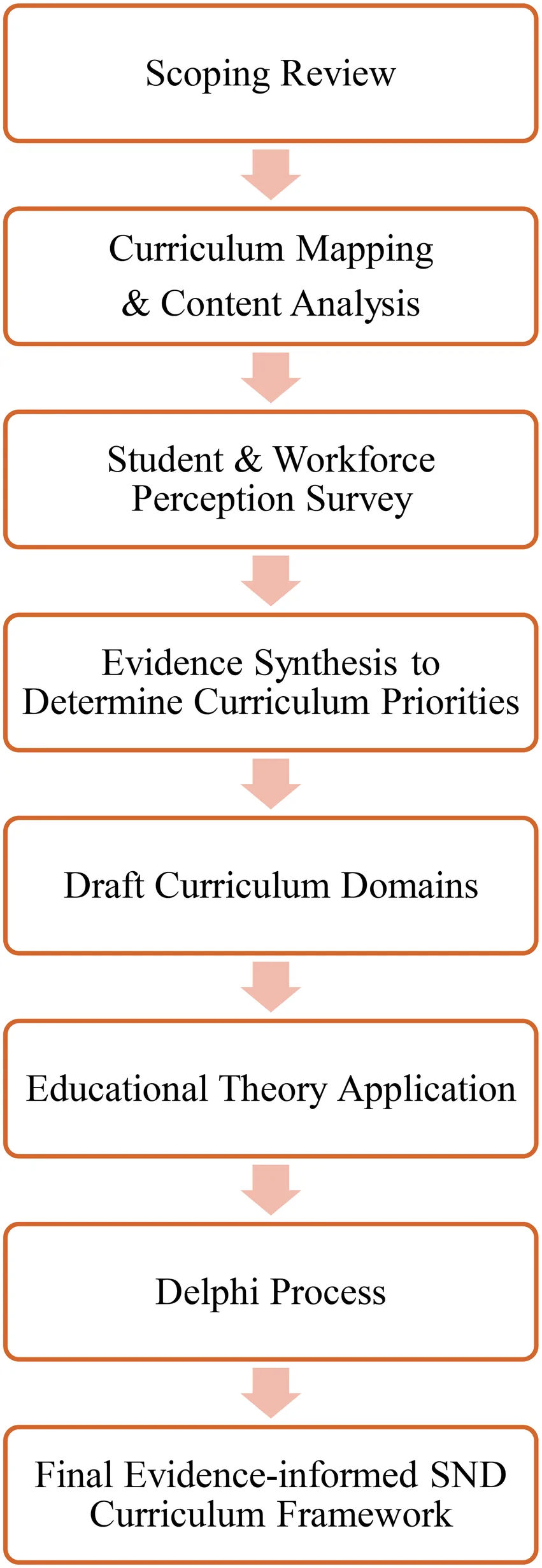
The development of the evidence-informed special needs dentistry curriculum framework.

Grounded in established educational principles, the framework emphasises the integration of theoretical knowledge with clinical experience, the development of communication and behaviour management skills, interprofessional collaboration, and person-centred care aligned with the social model of disability (8). It is intended to complement existing competency standards and international recommendations, including those proposed by the International Association for Disability and Oral Health (iADH) (12), while remaining adaptable across diverse educational contexts. This paper presents an evidence-informed and pedagogically grounded curriculum framework for SND that translates findings from a multi-phase research program into a practical and replicable model for integration into oral health therapy education. The framework aims to enhance educational preparedness by providing structured opportunities for learners to progressively develop the knowledge, clinical reasoning, and professional behaviours required for SND practice. Unlike the individual studies that informed its development, this paper focuses on translating empirical evidence into an integrated educational framework that supports curriculum design, implementation, and competency development. Strengthening SND education for OHTs has the potential to support a more confident and capable workforce, reduce reliance on specialist services, and improve access to equitable oral healthcare for people with disability (13).

For clarity, this manuscript distinguishes between preparedness, confidence, competence, and capability, as these constructs represent related but distinct aspects of workforce development. Preparedness refers to learners’ perceived readiness resulting from their educational experiences, whereas confidence reflects their self-belief in performing clinical tasks. Competence refers to the demonstrated ability to apply knowledge, skills, and professional behaviours safely and effectively in practice, while capability encompasses the capacity to adapt these competencies to increasingly complex and evolving clinical situations. Increased confidence is expected to develop alongside competence but is not regarded as a surrogate measure of clinical performance. The framework’s impact on learner capability and workforce outcomes should be evaluated following implementation.

## 2 Framework development process

The proposed SND curriculum represents the culmination of phases of research, with each phase contributing evidence that informed the curriculum design (Table 1). The initial phase comprised a scoping review examining barriers and facilitators influencing dental practitioners’ willingness to provide care for people with disability. This review consistently identified inadequate undergraduate education, limited clinical exposure, and low practitioner confidence as major barriers to providing care. Conversely, additional education and clinical opportunities, and supportive learning environments were recognised as important facilitators. These findings established the educational priorities for the framework and highlighted the need for structured, longitudinal learning experiences that progressively develop knowledge and clinical competence (9). The second phase involved curriculum mapping and content analysis of Australian oral health therapy programs. This identified substantial variation in the breadth, depth, and delivery of SND content across institutions, with many programs embedding disability-related content within broader subjects rather than delivering it systematically. These findings informed the development of comprehensive curriculum domains to promote consistency while maintaining flexibility for implementation across different educational settings (7, 10). The third phase explored perceptions of oral health students and the existing workforce regarding preparedness, confidence, and future training needs in SND. Participants consistently reported limited confidence in managing patients with disability despite expressing strong interest in further education and clinical experience. Communication, behavioural management, interdisciplinary collaboration, and supervised clinical opportunities emerged as priority areas requiring greater emphasis. These findings informed the learning outcomes, recommended learning activities, and assessment approaches incorporated throughout the framework (8).

**Table 1:** Translation of research findings into evidence-informed SND curriculum framework .

Research Phase	Key findings	Translation into curriculum framework
Scoping review	Identified inadequate education and limited supervised clinical experience as major barriers to practitioners' confidence and willingness to treat people with disability Education was consistently reported as a key facilitator of preparedness	Established the educational rationale for the framework by highlighting the need for greater emphasis on SND education and supervised clinical experiences
Curriculum mapping & content analysis	Demonstrated considerable variation in the quantity, timing, and breadth of SND content across Australian oral health programs, with inconsistent alignment to international learning outcomes	Informed the development of structured curriculum domains and learning outcomes to promote greater consistency across programs Informed the selection of feasible teaching, learning, and assessment strategies, while recognising implementation challenges within existing oral health programs
Student & workforce perception study	Reported variable confidence and low preparedness. Strong support was expressed for additional undergraduate and postgraduate education Communication, behaviour management, and clinical experience identified as priority areas	Guided the emphasis on communication skills, behaviour management, person-centred care, interprofessional collaboration, and increased supervised clinical learning throughout the curriculum
Delphi study	Experts reached consensus on curriculum domains, learning outcomes, learning activities, assessment tasks, and expected competency levels across undergraduate and postgraduate education	Refined and validated the curriculum framework to ensure educational relevance, feasibility, and applicability to oral health therapy curriculum

Evidence generated across these research phases was subsequently synthesised with additional curriculum and competency resources to inform the development of a preliminary framework for Delphi review. These resources included publicly available student handbooks from undergraduate oral health programs (e.g., Bachelor of Oral Health/Therapy) and postgraduate SND specialist training programs (e.g., Doctor of Clinical Dentistry), as well as undergraduate and postgraduate SND education guidelines developed by the International Association for Disability and Oral Health (iADH) and competency standards from the Australian Dental Council (12, 14, 15). Rather than adopting these sources directly, the research team undertook a thematic synthesis to identify recurring competency areas, group similar concepts, remove duplication, and ensure alignment with the scope of practice of OHTs. This process produced a preliminary curriculum framework comprising ten curriculum domains (Table 2) and associated learning outcomes. These sources were used as a structured foundation for expert review, as a starting point for the Delphi process to build on.

**Table 2:** Learning domains for Delphi process.

Domain	Title
1	The Scope of Special Needs Dentistry
2	Access and Barriers to Oral Health for People with Disability and Other Underserved Populations
3	Professional, Legal And Ethical Context Of Special Needs Dentistry (including consent matters)
4	Cultural Awareness and Sensitivities of Special Needs Dentistry Including Aboriginal and Torres Strait Islander Cultural Competency
5	Communication Skills in Special Needs Dentistry
6	Impact of Impairments, Disabilities and Systemic Conditions on Oral Health and Oral Function
7	Clinical Management of Patients Requiring Special Needs Dentistry
8	Clinical Pathways and Team Approach to Care
9	Dental Public Health and Oral Health Promotion
10	Clinical Research and Governance

## 3 Pedagogical framework

Following the development of the preliminary curriculum framework, established educational theories were applied to support the progressive acquisition of knowledge, clinical skills, and professional competencies. The framework adopts a learner-centred and competency-based approach, integrating theoretical knowledge with clinical application to enhance preparedness for real-world practice. To underpin this approach, the Spiral Curriculum was adopted as the overarching educational model (Figure 2). This model emphasises revisiting key concepts at increasing levels of complexity over time, enabling learners to progressively deepen their understanding (16). Such an approach is particularly relevant to SND education, where competency development requires the integration of knowledge, clinical experience, and attitudinal growth in managing patients with disability. In parallel, the framework is aligned with Miller’s Pyramid to support the translation of knowledge into clinical performance (17). This model conceptualises competency development across hierarchical levels, progressing from knowledge acquisition (“knows”) and application (“knows how”) to demonstration (“shows how”) and independent clinical practice (“does”) (18). This progression reinforces that learners are expected not only to acquire theoretical understanding but also to demonstrate competence in both simulated and real clinical environments.

**Figure 2:**
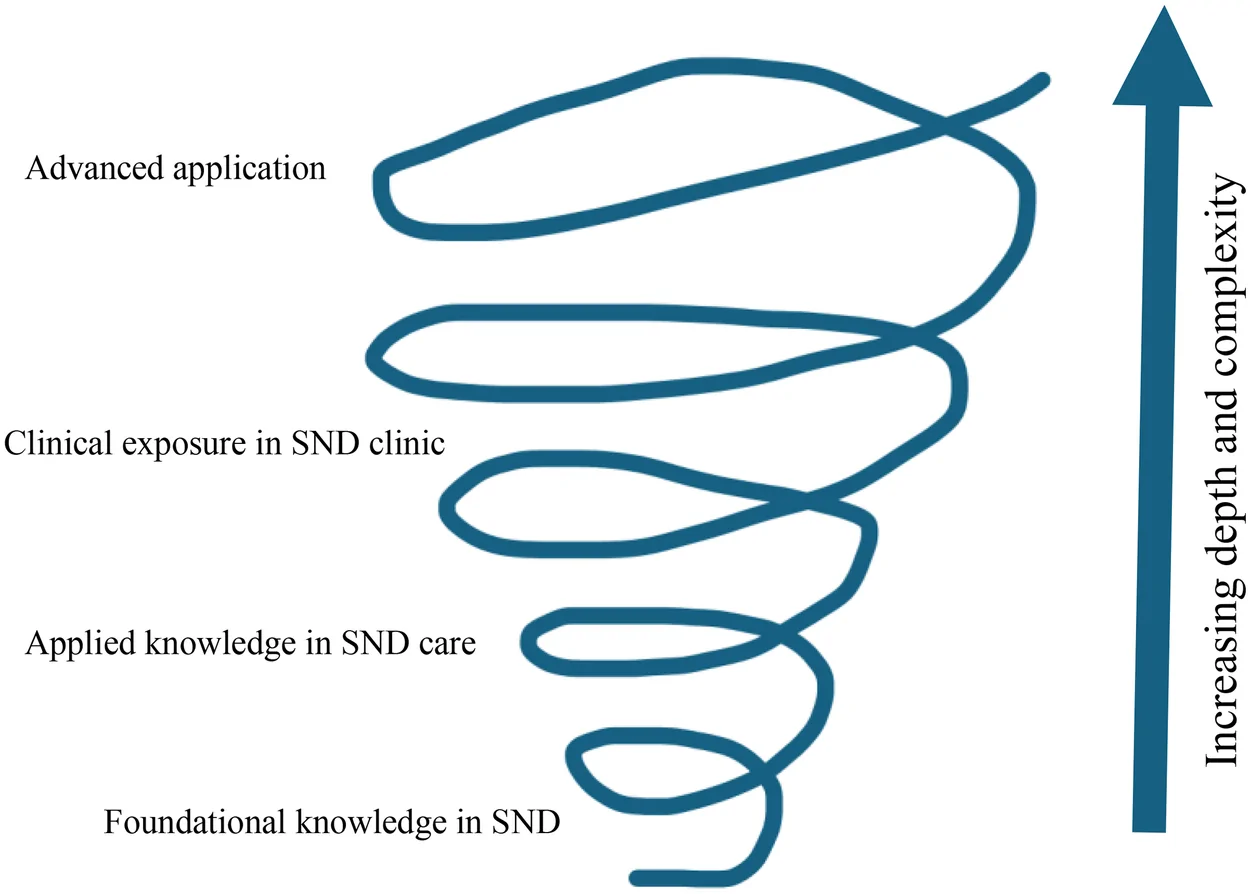
Illustrates the conceptual application of the spiral curriculum within SND, demonstrating how core knowledge and skills are revisited and expanded throughout the learning trajectory. (8) *Source: Special Needs Dentistry Training and Education for Preparedness in the Oral Health Therapy curriculum: Taking a STEP Forward. Reproduced with permission*.

Within the SND curriculum, learning outcomes are mapped across these levels to ensure alignment between teaching, learning, and assessment. Early stages of the program focus on foundational knowledge and awareness, while intermediate stages emphasise clinical reasoning and decision-making. Advanced stages support the demonstration of clinical skills in simulated environments and, ultimately, the provision of care in real-world settings (19). Table 3 illustrates an example of how each level of Miller’s Pyramid can be integrated into SND education and evaluated through appropriate assessment methods. This interplay of theory and practice is particularly vital in SND, where practitioners must navigate complex patient needs, ethical considerations, and individualised care approach, considering that every patient is unique (20). This ensures that educational outcomes extend beyond theoretical understanding to the development of practical clinical capabilities. Together, the integration of the spiral curriculum and competency-based frameworks provides a coherent pedagogical foundation to support education in SND.

**Table 3:** Presents examples of how learning outcomes, action verbs, and assessment strategies are aligned with the different levels of miller's pyramid within the SND context. This structured alignment facilitates a coherent progression from knowledge acquisition to clinical competence and performance.

Perceived phases	Levels (Miller's pyramid)	Examples of action verbs on learning outcomes	Examples in SND context	Assessment examples
Early	Knows	Explain, understand, recognise, describe, define, identify, outline, discuss, describe key concepts, summarise	Understands foundational SND concepts	Written exams, MCQs, short-answer questions
Mid	Knows how	Explore, integrate, assess, facilitate, evaluate, differentiate, analyse, sort, compare, contrast, interpret, organise	Applies knowledge to clinical decision-making in SND contexts	Case studies, clinical simulations, and extended multiple-choice questions.
Transition-to-practice	Shows how	Implement, obtain, engage, advocate, critically reflect, use, develop, manage, provide, practice, adopt, critically review, plan, perform, apply, diagnose, demonstrate, analyse, critique, create, design, debate	Demonstrates SND skills in a simulated environment	OSCEs and simulated patient encounters
Practice	Does		Provides competent SND care in real clinical settings	Direct observation in clinical settings, workplace-based assessments, and performance-based evaluations.

## 4 Curriculum design

The preliminary framework was subsequently refined through a multi-round Delphi study involving experts in SND, oral health therapy, dental education, curriculum development, and related disciplines. Experts reviewed the proposed curriculum domain and learning outcomes, suggested new learning outcomes, providing qualitative feedback and quantitative ratings regarding relevance, clarity, and importance. Following each Delphi round, responses were analysed and the framework was revised to reflect panel feedback. Items that did not achieve consensus were either modified and redistributed for further review, or removed (11).

The curriculum framework was designed as an integrated competency-based model in which each curriculum component contributes to the progressive learning in SND. The curriculum domains define the essential areas of practice, while the learning outcomes specify the competencies expected at different stages of training. These outcomes inform the selection of teaching and learning activities, which provide opportunities for learners to acquire, apply, and consolidate knowledge through clinical experiences. Assessment tasks are constructively aligned with the intended learning outcomes to evaluate competency across multiple stages of learning. These curriculum components interact to support the progressive development of competence. Learners revisit core concepts through increasingly complex learning experiences while progressing from foundational knowledge to independent clinical practice (Table 4).

**Table 4:** Interaction of curriculum components to support progressive competency development in SND.

Curriculum component	Function
Domains	Define what should be learned
Learning outcomes	Define what learners should achieve
Learning activities	Provide how learners develop competencies
Assessment tasks	Evaluates whether competencies have been achieved
Spiral curriculum	Explains when and how competencies are revisited and strengthened
Miller's Pyramid	Explains how learners progress from knowledge to clinical practice

### 4.1 Learning outcomes

Learning outcomes are aligned with each domain and are structured using Bloom’s Taxonomy and action verbs to differentiate levels of learning (21). At foundational levels, outcomes focus on building knowledge and awareness of disability, health disparities, and patient-centred care principles. As learning progresses, outcomes emphasise the application of knowledge in clinical contexts, including risk assessment, treatment planning, behaviour control, and communication with patients and caregivers. At more advanced levels, learning outcomes incorporate clinical decision-making, adaptability in managing complex cases, and collaboration with interdisciplinary teams. The sequencing of learning outcomes aligns with the principles that ensure the learners transition from understanding theoretical concepts to demonstrating competence in clinical environments.

### 4.2 Learning activities

Suggested learning activities are provided as a supplementary guide to support the achievement of learning outcomes and may be adapted to suit institutional contexts and resources. These activities are designed to promote active learning, clinical reasoning, and the development of practical competencies relevant to SND practice.

#### 4.2.1 Teaching and learning strategies

A blended pedagogical approach is recommended to support the delivery of the SND curriculum. Teaching strategies include didactic lectures, case-based learning, simulation activities, and reflective practice. Case-based discussions and problem-based learning are used to contextualise theoretical knowledge and encourage critical thinking (22), while simulation-based training provides a safe environment for students to develop communication and behavioural management skills (23).

Clinical learning is a central component of the framework, with supervised exposure to patients with disability integrated throughout the program. Learners should be introduced to SND concepts early in their training and be provided with sufficient opportunities to engage in clinical care during their curriculum, to build confidence and competence over time (19, 24). Clinical exposure may include rotations in special care clinics, community outreach programs, and integrated care settings. These experiences provide opportunities for students to observe, assist, and participate in the delivery of care for people with disability (25). Reflective activities, such as journaling and debriefing sessions, should also be incorporated to support the development of professional judgement and self-awareness (26).

### 4.3 Assessment tasks

Assessment methods are aligned with competency-based education to evaluate learning across multiple levels. Knowledge acquisition may be assessed through written examinations and case-based assessments, while clinical competence is evaluated through objective structured clinical examinations (OSCEs), simulation exercises, and workplace-based assessments (10, 12). Performance in real clinical settings is assessed through supervised clinical evaluations, with feedback provided to support ongoing development (27). Reflective assessments can also be included to encourage critical thinking and self-evaluation (28). This multi-modal assessment approach ensures that learners are assessed not only on what they know, but also on their ability to apply knowledge in practice.

### 4.4 Postgraduate component

The proposed SND curriculum framework is primarily designed for integration within undergraduate oral health therapy programs, with extension to postgraduate and continuing professional development contexts. The undergraduate component represents the introductory level of SND education and aims to ensure that all graduating oral health therapists possess the essential knowledge, skills, and professional attitudes required to provide safe and inclusive care for people with disability. Undergraduate competencies focus on developing awareness of disability and health inequities, effective communication, preventive care approaches, basic clinical adaptation, behavioural support strategies, and collaboration with patients, caregivers, and the broader healthcare team. These competencies are considered essential for entry-level practice and reflect the expectation that all oral health professionals should be prepared to contribute to equitable oral healthcare. In contrast, the postgraduate component was designed to represent advanced competency development for practitioners seeking further training and professional progression in SND. While undergraduate education focuses on preparedness for general clinical practice, postgraduate competencies extend towards the management of more complex cases, advanced treatment planning, interdisciplinary care coordination, leadership in care delivery, advocacy, education, research, and engagement with broader health systems. These competencies require greater clinical experience and a deeper understanding of the complex social, medical, and systemic factors influencing care delivery. Such competencies may not yet be embedded within structured programs; their inclusion ensures that they are not overlooked in future curriculum development.

While the immediate focus is on preparing entry-level practitioners, the inclusion of a postgraduate component reflects a longer-term vision for workforce development. This also aligns with emerging interest among the oral health workforce in further education and skill development in SND (8). At present, formal postgraduate training pathways in SND for the oral health workforce remain non-existent in Australia and internationally. However, the profession is evolving, with increasing recognition of the need for career progression opportunities and advanced training pathways. The inclusion of postgraduate competencies ensures that higher-level competencies are identified and retained within the broader curriculum framework while allowing institutions and professional bodies to adapt implementation according to future workforce needs. This reflects the principles of progressive competency development, where learners transition from foundational knowledge and supervised clinical practice towards greater autonomy, complexity, and leadership in care delivery. By incorporating both undergraduate and postgraduate pathways, the framework provides a comprehensive educational continuum that supports the development of a capable oral health workforce.

## 5 Discussion

Recent national policy initiatives in Australia have emphasised the importance of embedding disability-related competencies across all stages of health professional education. Frameworks guiding workforce development increasingly advocate for the inclusion of disability-inclusive training within undergraduate curricula, as well as across postgraduate education and continuing professional development (29). These directions reflect a broader shift towards ensuring that health professionals are equipped to deliver equitable and accessible care to populations experiencing systemic barriers. Within oral health, accreditation bodies have similarly highlighted the need for graduates to demonstrate competence in managing priority populations at higher risk of poor oral health outcomes, including people with disability (15). Contemporary capability frameworks, such as those focused on intellectual disability, further reinforce the importance of equipping future practitioners with the knowledge, skills, and professional behaviours required to provide safe and appropriate care (30). Collectively, these developments stress the need for structured, competency-based approaches to disability-inclusive education.

The curriculum framework presented in this study responds to these priorities by offering a discipline-specific model for integrating SND into oral health therapy education. By focusing on the development of knowledge, clinical exposure, and practitioner confidence, the framework addresses key barriers that have been consistently identified within the oral health workforce. Embedding these competencies at the undergraduate level is particularly important, as it ensures that all graduates, not only those pursuing further training, are prepared to provide care for people with disability. The spiral approach offers several educational advantages within SND education. Repeated engagement with core concepts may enhance knowledge retention, reinforce clinical reasoning, and support the gradual development of competence and confidence. Progressive exposure to increasingly complex scenarios allows learners to consolidate earlier learning while integrating new knowledge and skills within clinical contexts. This is particularly important in SND, where students may initially experience uncertainty or anxiety when managing patients with disability. This way, learners are provided with repeated opportunities to strengthen both technical and professional judgement skills.

A key strength of the proposed framework is its emphasis on integration as opposed to expansion of an already crowded curriculum content. Rather than introducing additional standalone units, the framework is intended to support curriculum mapping, incorporating SND-related learning outcomes within existing subject areas, including clinical practice, public health, ethics, and communication. This approach enables programs to enhance disability-related training without increasing curriculum burden, while maintaining alignment with accreditation requirements (11). The framework also adopts a staged approach to competency development and is designed to be adaptable, allowing institutions to tailor implementation based on local resources, clinical placement availability, and workforce needs. Another important consideration is the alignment between learning outcomes, teaching strategies, and assessment. Although a single assessment model was not prescribed, the inclusion of suggested learning activities and assessment approaches provides practical guidance for educators. This allows institutions flexibility while maintaining constructive alignment and facilitates the translation of curriculum design into measurable educational outcomes.

Implementation of the framework will inevitably vary according to institutional resources, clinical placement availability, and access to specialised services. Where authentic clinical experiences are limited, alternative educational approaches such as simulation, virtual patient cases, interprofessional education, community outreach, and structured reflective practice may provide meaningful opportunities for competency development (10). Simulation-based activities, including standardised patient encounters and scenario-based training, allow learners to practise communication strategies, behavioural management techniques, and clinical decision-making in a safe environment before engaging with patients (23). Virtual case discussions and digital learning resources can further broaden exposure to complex clinical scenarios, while partnerships with disability advocates, carers, allied health professionals, and community organisations may enhance understanding of the lived experiences of people with disability and strengthen person-centred care (31). Although these strategies should complement rather than replace direct patient care, they provide realistic alternatives that maintain alignment with the intended competencies of the curriculum framework when clinical capacity is constrained.

As a newly developed framework, its application in educational settings has not yet been formally evaluated. The framework was developed within the Australian context, where oral health therapists undertake a dual role encompassing both dental hygiene and therapy. This informed the inclusion of competencies related to preventive care, restorative treatment, and broader contributions to community-based oral health services. While the framework may be applicable to other oral health programs, including those focused solely on dental hygiene or therapy, adaptation would be required to ensure alignment with different scopes of practice and regulatory environments. Nevertheless, it provides a structured foundation that can support the integration of disability-inclusive competencies across a range of educational contexts.

Future research should focus on evaluating the implementation of the framework in practice. Pilot studies within oral health programs would enable assessment of their feasibility, acceptability, and impact on student learning and clinical preparedness. Such work would also help determine whether the proposed progression between undergraduate and postgraduate competencies aligns with institutional capabilities and learner needs. Further investigation into the experiences of practising oral health therapists may also provide valuable insights into ongoing workforce needs, particularly in relation to confidence and perceived competence in managing patients with disability. These findings could inform future refinement of the framework and support its development as a responsive educational model.

Despite the potential benefits of implementing the framework, some of the practical challenges should be acknowledged. Effective delivery requires strong vertical integration across subjects, access to appropriate clinical settings, and faculty calibration to ensure consistency in teaching and assessment practices. Successful implementation will also depend on faculty preparedness. Educators require sufficient knowledge, confidence and experience to facilitate disability-inclusive learning, supervise students in clinical environments, and apply assessment criteria consistently. Faculty development initiatives, calibration activities, and partnerships with clinicians experienced in SND may therefore be necessary to support consistent implementation across institutions. Resource limitations present an additional consideration. Universities differ in curriculum capacity, staffing, simulation facilities, and access to disability services, requiring flexible approaches that integrate SND learning within existing subjects rather than adding substantial new curriculum content. Access to authentic clinical placements may also vary according to specialist service availability and supervisor capacity. Where repeated supervised exposure is not feasible, complementary approaches such as simulation, virtual case discussions, community outreach, and interprofessional learning may help reinforce competency development. Finally, assessment of SND competence presents practical challenges because professional behaviours, communication, and clinical adaptability are best evaluated through direct observation in authentic settings. A combination of workplace-based assessments, OSCEs, simulation activities, and reflective practice may provide a more feasible and comprehensive approach to evaluating learner progression.

The contribution of this study extends beyond reporting the findings of individual research phases. Previous publications from this research program examined specific aspects of SND education, including barriers to care, existing curriculum content, workforce preparedness, and expert consensus regarding curriculum content. In contrast, the present manuscript synthesises these complementary sources of evidence into a coherent educational framework informed by established pedagogical theory. By explicitly linking empirical findings with curriculum design, competency development, teaching strategies, assessment approaches, and implementation considerations, this study provides educators with a practical model that can be adapted within oral health therapy programs.

## 6 Conclusion

The curriculum framework supports a staged progression of learning, beginning with foundational knowledge in the early stages of learning, followed by simulation-based skill development, and demonstrating patient care in supervised clinical practice. This progression enables learners to gradually build competence and confidence, aligning with principles of scaffolded learning and preparing graduates for independent practice in diverse clinical settings. Taken together, these elements support the development of a competent and confident oral health workforce capable of delivering inclusive, person-centred care.

## Author's Note

This manuscript presents an educational framework developed through the synthesis of findings from a multi-phase research program. No new human participant data were collected or analysed for the purpose of this manuscript. The underlying studies that informed the framework, including the workforce and student perception studies and Delphi consensus study, received ethical approval prior to participant recruitment. Findings from these previously approved studies have been reported separately, and this manuscript focuses on the integration of these findings into an evidenceinformed curriculum framework and its pedagogical and implementation considerations.

The final document, “Undergraduate and Postgraduate Special Needs Dentistry Curricula for Oral Health Therapists” can be downloaded from the Appendices in the Supplementary material.

## Data Availability

The original contributions presented in the study are included in the article/Supplementary material, further inquiries can be directed to the corresponding author.
