# Supplementary material for: Special needs dentistry training and education for preparedness in the oral health therapy curriculum
Source: Front Oral Health. 2026 Sep 24;7:1896214. doi: 10.3389/froh.2026.1896214 (PMC13645591; doi:10.3389/froh.2026.1896214)
Supplement: Supplementary file 1 [file Supplementaryfile1.pdf]

Undergraduate and Postgraduate Special Needs Dentistry Curricula for Oral Health Therapists

THE UNIVERSITY OF SYDNEY

Statement of Intent

With the global increase in the number of people living with disability, there is a growing and largely unmet demand for oral health care within this population. The Oral Health Therapy workforce is well-positioned to address this need, given their knowledge and expertise in dental disease prevention and oral health promotion.[1]

Research shows that dental practitioners who reported positive experiences with their Special Needs Dentistry (SND) training are more confident and more willing to provide dental care to patients with disability.[2] Evidence also indicates a strong correlation between adequate clinical exposure during undergraduate training and practitioners' likelihood to provide care to people with disability in their future practice.[3] Despite this, the current literature highlights both insufficient educational opportunities and significant variation in SND educational training within undergraduate dental curricula.[4]

"Education is a gateway to improved equality in dental care for people with disability" - O'Rourke et al.[5]

A key focus of Australia's National Roadmap for Improving the Health of People with Disability is the integration of disability health capability frameworks into education. This includes embedding disability health training across all levels of health practitioner education, from entry-level to postgraduate programs.[6] It is therefore critical that SND content, both theoretical and clinical, be sufficiently integrated into oral health curricula to ensure graduates are equipped to provide inclusive, equitable care for people with disability.

In response, we initiated the development of this curriculum guidance document through a Delphi process involving expert panellists from multiple countries. This document outlines learning domains and outcomes, along with examples of learning activities and assessment tasks. In alignment with the *Convention on the Rights of Persons with Disabilities (CRPD)*, which asserts the right of people with disability to "the enjoyment of the highest attainable standard of health without discrimination on the basis of disability," this document aims to establish a standardised and comprehensive curriculum framework as an essential educational strategy to enhance the competence and confidence of the OHT workforce in SND—thereby helping reduce inequities in the oral health care of people with disability.

Dorothy Koh, A/Prof Archana Pradhan, A/Prof Melanie Aley, A/Prof Delyse Leadbeatter, Prof Woosung Sohn

Content

Statement of Intent	2
Authors and Acknowledgements	5
Glossary	6
Learning Domains in Special Needs Dentistry	7
Domain 1: The Scope of Special Needs Dentistry	8
Domain 2: Access and Barriers to Oral Health for People with Disability and Other Underserved Populations	10
Domain 3: Professional, Legal And Ethical Context Of Special Needs Dentistry (including consent matters)	12
Domain 4: Cultural Awareness and Sensitivities of Special Needs Dentistry Including Aboriginal and Torres Strait Islander Cultural Competency	14
Domain 5: Communication Skills in Special Needs Dentistry	16
Domain 6: Impact of Impairments, Disabilities and Systemic Conditions on Oral Health and Oral Function	18
Domain 7: Clinical Management of Patients Requiring Special Needs Dentistry	20
Domain 8: Clinical Pathways and Team Approach to Care	22
Domain 9: Dental Public Health and Oral Health Promotion	24
Domain 10: Clinical Research and Governance	26
References	28

Authors and Acknowledgements

This document was prepared by Dorothy Koh, A/Prof Archana Pradhan, A/Prof Melanie Aley, A/Prof Delyse Leadbeatter and Prof Woosung Sohn. This document can be freely adapted and used for teaching and training purposes.

The authors would like to thank the Delphi panel members for their valuable contribution to the creation of this document:

Dr Mathew AWT Lim, Australia

Dr Keng Soon Yeoh, Australia

Dr Troy Longbottom, Australia

Prof Hanny Calache, Australia

Ms Karen L Lansdown, New Zealand

Dr Denise Faulks, France

A/Prof Masita Mandasari, Indonesia

Mr Samuel D Carrington, New Zealand

Prof June H Nunn, Republic of Ireland

Dr Caoimhin Mac Giolla Phadraig, Republic of Ireland

Assistant Prof Clive Friedman, Canada

Dr Kadambari Rawal, United States

Dr Jessica Francis, Malaysia

Dr Ann Spolarich, United States

Prof Emma Bingham, United Kingdom

Ms Ceara Cleary, Republic of Ireland

Glossary

Terms	Definition
Special Needs Dentistry (SND)	The Royal Australasian College of Dental Surgeons (RACDS) defines SND as "the branch of dentistry that is concerned with the oral health care of people with an intellectual disability or medical, physical, and/or psychiatric conditions that require special methods or techniques to prevent or treat oral health problems or where such conditions necessitate special dental treatment plans.[7]<br><br>In line with the Australian Government's inclusive language guidelines, this document adopts more inclusive terminology by replacing the term "special needs" with "disability", and including person-first language when referring to people with disability.[8]
Interprofessional	According to the World Health Organisation (WHO), interprofessional education (IPE) occurs when students from two or more professions learn about, from, and with each other to enable effective collaboration and improve health outcomes.[9]<br><br>The Australian Dental Council (ADC) defines interprofessional collaborative practice as multiple health workers from different backgrounds working together with patients, families, and communities to deliver high-quality care.[10]
Intersectoral	A broader approach that goes beyond healthcare, involving collaboration between different sectors.[11]

Learning Domains in Special Needs Dentistry

Domain	Title
1	The Scope of Special Needs Dentistry
2	Access and Barriers to Oral Health for People with Disability and Other Underserved Populations
3	Professional, Legal And Ethical Context Of Special Needs Dentistry (including consent matters)
4	Cultural Awareness and Sensitivities of Special Needs Dentistry Including Aboriginal and Torres Strait Islander Cultural Competency
5	Communication Skills in Special Needs Dentistry
6	Impact of Impairments, Disabilities and Systemic Conditions on Oral Health and Oral Function
7	Clinical Management of Patients Requiring Special Needs Dentistry
8	Clinical Pathways and Team Approach to Care
9	Dental Public Health and Oral Health Promotion
10	Clinical Research and Governance

1. The Scope of Special Needs Dentistry

<table>
<tr><th>Learning outcomes</th><th>Undergraduate</th><th>Postgraduate</th></tr>
<tr><td></td><td>Explain the need for and apply the use of appropriate terminology.
Understand the need and growth of the emerging specialty of SND.
Demonstrate an understanding of life course approach to oral health and disability.
Describe the key concepts, epidemiology, and classification related to human function, impairments, disability, and health.</td><td>Integrate appropriate terminology in clinical discussions and patient care.
Apply the International Classification of Functioning, Disability and Health (ICF) framework in clinical decision-making.
Apply an advanced understanding of life course approach of SND.
Interpret and apply key concepts, epidemiology, and classification systems in patient care planning.</td></tr>
<tr><td>Learning activities</td><td colspan="2">Case-based learning and simulated-based training using virtual reality (VR) and artificial intelligence (AI).
Group-based case studies and discussions with healthcare professionals from different fields.
Observation in specialty clinics (e.g., aged care, disability services, medical specialties).
Field visits to organisations supporting people with disability and aged-care facilities.
Real-time patient interviews in various settings (e.g., long-term care facilities, nursing homes, home care).
Panel discussions with people with disability sharing their experiences with access barriers.
Sensory walkthrough exercises – Simulating the experience of a patient with sensory processing issues in a dental clinic.
Role-playing as people with disability (e.g., using ankle weights, sunglasses, noise-cancelling headphones) to simulate physical and cognitive impairments.
Developing new online learning materials in collaboration with people with lived experiences.
Reflective journals after clinical exposure or patient interactions.</td></tr>
</table>

	• Critical appraisal of healthcare models through structured reflection exercises.
**Assessment tasks**	• Case-based oral and written assessments focusing on roles, responsibilities, and scope of practice.<br>• Reflective essays/journals on student experiences in SND, including personal learning reflections, how experiences influence future care provision.<br>• Essay topics exploring aspects of SND, ethical considerations, and patient management strategies.<br>• Written exams testing core theoretical understanding of SND principles.<br>• Clinical case presentations where students present a case they have managed, requiring detailed documentation, pattern recognition in clinical care, and potential publication in peer-reviewed journals.<br>• Student-led seminars and case presentations on SND topics, with peer discussions and Q&A sessions.

2. Access and Barriers to Oral Health for People with Disability and Other Underserved Populations

Learning outcomes	Undergraduate	Postgraduate
	1. Identify social, environmental and attitudinal barriers and facilitators to oral healthcare services.<br>2. Apply knowledge of the social determinants of health to identify health inequalities and their implications on oral health.<br>3. Apply an understanding of barriers and facilitators to oral health in patient assessment, prevention, treatment planning, and the provision of oral health care.<br>4. Identify external key stakeholders to overcome access barriers.<br>5. Identify different types and sources of disability support funding and advocacy.	1. Critically assess social, environmental, and attitudinal barriers and facilitators to oral healthcare services, incorporating real-world case studies.<br>2. Analyse the impact of social determinants of health on oral health inequalities and develop strategies to address them in clinical practice.<br>3. Implement and justify tailored strategies to address barriers and improve oral health outcomes, considering ethical and practical implications.<br>4. Explore external key stakeholders to collaborate with in order to improve access to oral health care.<br>5. Explore opportunities and application processes for funding aimed at supporting people with disability.
**Learning activities**	• Collaborating with social workers to understand and address access barriers<br>• Mapping barriers and facilitators to identify social, environmental, and attitudinal factors affecting dental care access for people with disability<br>• Disability equality and inclusion training to enhance student understanding of challenges faced by people with disability	

	• Field visits to NGOs, nursing homes, or disability associations to observe real-world care settings<br>• Home visits to engage directly with families and understand the lived experiences of people with disability<br>• Workshops with people with lived experience, focusing on discriminatory behaviours and how oral health therapists can remove barriers<br>• Case studies analysing social determinants of health, examining health inequalities and their impact on dental care access<br>• Advocacy campaign projects, where students develop campaigns (e.g., policy briefs, blog posts, social media initiatives) to raise awareness about oral health needs in disability care.
**Assessment tasks**	• Case scenarios on barriers faced by people with disability and strategies to overcome them.<br>• Simulated patient record analysis, students identify inappropriate terminology, assess determinants of poor oral health, and develop an improved treatment plan with preventive strategies.<br>• Reflective essays and journals, where students analyse their experiences related to disability access and care.<br>• Portfolio-based assessments, documenting learning, experiences, and strategies for improving care.<br>• Oral health needs assessment project, requiring students to assess a high-disability-prevalence community and propose public health initiatives.<br>• Written exams testing theoretical understanding of disability barriers and solutions.

3. Professional, Legal And Ethical Context Of Special Needs Dentistry (including consent matters)

<table>
<tr><th>Learning outcomes</th><th>Undergraduate</th><th>Postgraduate</th></tr>
<tr><td></td><td>1. Explain the social, legal, and ethical considerations related to supported decision-making.
2. Demonstrate proficiency in evaluating an individual’s capacity to provide consent.
3. Recognise the role of the family members, carers and the relevant government agencies in assisting decision-making processes.
4. Facilitate the process of obtaining informed consent for individuals who are unable to consent on their own behalf, ensuring it adheres to legal and ethical standards.</td><td>1. Evaluate the role of family members, carers, and relevant government agencies in assisted decision-making, integrating ethical frameworks.
2. Describe the scope of a public fiduciary, a medical power of attorney, and a legal guardian and their roles in providing consent on behalf of another individual.
3. Obtain informed consent for patients who are unable to consent on their own behalf, ensuring compliance with legal and ethical standards.</td></tr>
<tr><td>Learning activities</td><td colspan="2">• Guest lectures and webinars with legal professionals (lawyers, Civil Administrative Tribunal, Office of Public Advocate, ADA representatives) discussing consent laws, guardianship, and decision-making complexities.
• Review of the Mental Capacity Act and Mental Health Treatment Orders to understand legal obligations in dental care.
• Critique of the Dental Practice Act with discussions on supervision requirements in alternative care settings.</td></tr>
</table>

	• Case-based problem-based learning (PBL) discussions to explore complex consent issues and ethical dilemmas.<br>• Case scenario discussions on informed consent, guardianship disputes, and decision-making in complex situations.<br>• Role-playing exercises on assisted decision-making processes, motivational interviewing techniques, and ensuring patient comprehension of consent.<br>• SND clinics and domiciliary care to practice real-world consent processes in different care settings.<br>• Morbidity & Mortality (M&M) grand rounds exercises discussing real-life cases, treatment failures, and improvements.<br>• Interviews with caregivers and families who have made significant healthcare decisions to understand their perspectives.<br>• Lectures from social service professionals on supporting people with disability in healthcare decision-making.
**Assessment tasks**	• MCQs and computer-based tests evaluating students' understanding of consent laws and guardianship.<br>• Written exams and legislative research papers exploring the legal system and ethical considerations in obtaining consent for dental treatment.<br>• Case scenarios requiring students to identify and apply legislation to different consent situations.<br>• Simulated patient records and consent scenarios requiring students to assess cognition, apply legal principles, and document consent procedures.<br>• Reflective essays and portfolio submissions where students describe their interactions with people with disability and the legal challenges they faced.

4. Cultural Awareness and Sensitivities of Special Needs Dentistry Including Aboriginal and Torres Straits Islander Cultural Competency

Learning outcomes	Undergraduate	Postgraduate
	1. Demonstrate awareness of one's own cultural values and how they relate to other communities, families and individuals.<br>2. Understand principles of cultural safety, which involves creating a respectful and inclusive environment that acknowledges health beliefs and lifestyles, and respects cultural diversity.<br>3. Demonstrate an understanding of, and respect for, Australian Aboriginal and Torres Strait Islander values, culture and knowledge.<br>4. Engage in a respectful and reflective manner, employing culturally relevant, safe, and sensitive communication strategies to facilitate relationships.	1. Critically reflect on their own cultural identity and applying this awareness to interactions with diverse communities, families, and individuals.<br>2. Advocate for inclusive oral healthcare by fostering culturally responsive attitudes and promoting equity in healthcare delivery.<br>3. Demonstrate leadership in fostering culturally safe clinical environments by implementing best practices in Aboriginal and Torres Strait Islander healthcare.<br>4. Implement culturally tailored health promotion activities and community engagement initiatives that incorporate the traditional practices of Aboriginal and Torres Strait Islanders, to improve oral health literacy, inequalities and outcomes.
**Learning activities**	• Yarning circles with Aboriginal and Torres Strait Islander communities to discuss oral health challenges and build relationships.<br>• Service-learning in SND clinics and culturally diverse communities, providing care and deliver health education and services.<br>• Guest lectures from advocacy groups, Indigenous elders, and community leaders to provide firsthand insights into cultural values, traditions, and health barriers.	

	• Collaboration with medical interpreters to address communication barriers in healthcare.<br>• Cultural safety training modules and interdisciplinary case discussions to explore patient-centred care in diverse settings.<br>• Reflective journals to encourage self-awareness and critical thinking about cultural competence and patient interactions.<br>• Student presentations on health inequalities faced by Aboriginal and Torres Strait Islander and other minority communities, analysing root causes and proposing solutions.<br>• Workshops exploring lived experiences of marginalised groups, highlighting barriers to healthcare access and strategies for inclusive care.
**Assessment tasks**	• Cultural awareness training followed by an exam to reinforce key concepts.<br>• Essay exploring challenges in dental care for Aboriginal and Torres Strait Islander communities, addressing barriers, cultural considerations, and specific management strategies.<br>• Oral presentation where students propose oral health promotion strategies for an Aboriginal and Torres Strait Islander health centre.<br>• Health promotion campaign development, such as posters, brochures, or social media initiatives, tailored to ATSI communities.

5. Communication Skills in Special Needs Dentistry

Learning outcomes	Undergraduate	Postgraduate
	1. Use and reflect on culturally sensitive and inclusive language with patients, colleagues and carers with lived experience, to obtain an adequate medical and social history.<br>2. Develop adequate communication skills and use appropriate methods of communication, including supportive decision-making strategies, to engage in meaningful interaction based on patients' specific needs.<br>3. Identify and describe assistive communication technologies, including augmentative and alternative communication (AAC) methods.	1. Evaluate and reflect on culturally sensitive and inclusive communication strategies to enhance patient engagement and intersectoral collaboration.<br>2. Integrate and assess the effectiveness of augmented communication methods and shared decision-making approaches in clinical practice.<br>3. Critically reflect on patient narratives and lived experiences to shape communication strategies that promote patient autonomy and person-centred care.
**Learning activities**	• Clinical placement in SND units for observation and hands-on experience.<br>• Observational visits with specialists in SND and discussions with caregivers about effective communication strategies.<br>• Role-play exercises, including using cue cards to guide communication in simulated patient interactions. Students acting as people with disability (e.g., mute, deaf, visually impaired) to develop empathy and problem-solving skills.<br>• Workshops on augmentative and alternative communication (AAC) tools, including picture boards and apps (e.g., Proloquo2Go).	

	• Basic sign language training to improve interactions with non-verbal and hearing-impaired patients.<br>• Analysis of real-world patient narratives, identifying effective and ineffective communication strategies.<br>• Lived experience sessions with individuals who have disability, allowing students to gain firsthand perspectives.
**Assessment tasks**	• Direct observation of patient-clinician interaction and clinical staff's feedback on student communication.<br>• Case-based scenarios and clinical practice to assess practical knowledge and communication strategies.<br>• Reflection papers on student experiences with disabilities to develop empathy and self-awareness.<br>• Oral presentations on communication technologies and patient-centred strategies.

6. Impact of Impairments, Disabilities and Systemic Conditions on Oral Health and Oral Function

Learning outcomes	Undergraduate	Postgraduate
	1. Describe key concepts of health, disabilities and systemic conditions.<br>2. Explain the relationship between systemic and oral health, oral health related quality of life and their implications in the dental management.<br>3. Recognise the complexities of managing patients with a range of disability, social and medical issues, and understand the connection between general health and oral health.<br>4. Identify the different available behavioural support strategies and justify their use within the scope of practice.<br>5. Differentiate the characteristic oral manifestations of specific conditions.<br>6. Identify the impact of risk factors, systemic disease and medications on oral health and treatment planning.	1. Analyse key concepts of health, disabilities, and systemic conditions, applying them to advanced clinical contexts.<br>2. Evaluate the relationship between systemic and oral health, demonstrating its impact on oral health-related quality of life and clinical decision-making.<br>3. Apply knowledge of complex medical, social, and psychological factors to optimise patient-centred oral health care.<br>4. Implement evidence-based behavioural support strategies to enhance patient cooperation and improve oral health outcomes in clinical settings.<br>5. Manage the oral manifestations of specific conditions, incorporating clinical reasoning and interprofessional collaboration.<br>6. Develop tailored, risk-based oral health management plans, considering long-term outcomes in various clinical, aged care and home settings.
**Learning activities**	• Shadowing families and professionals in paediatrics, gerontology, and other specialist clinics.	

	• Problem-based learning with case scenarios to develop research and management skills.<br>• Detailed case studies of patients with systemic conditions to assess oral health impacts.<br>• Case study analysis and OHRQoL survey administration to evaluate the impact of disabilities on oral health.<br>• Writing care plans for selected medical conditions or disabilities, sharing findings as clinical guidance.
**Assessment tasks**	• Medical history-taking assessments, focusing on accuracy and potential oral manifestations.<br>• Student-made treatment plans based on standardised patient scenarios.<br>• Risk assessment and care plan writing based on medical conditions or disabilities.<br>• Oral examinations requiring modifications to treatment plans based on patient scenarios.<br>• Oral presentations where students share a patient's story and how impairments affected their dental care access and treatment, and investigate service delivery models for people with disability and aged-care residents through online research and interviews.

7. Clinical Management of Patients Requiring Special Needs Dentistry

Learning outcomes	Undergraduate	Postgraduate
	1. Demonstrate basic life support skills and management of medical emergencies including gerodontics. according to local protocols and guidelines.<br>2. Describe the factors (medical, social, cultural and environmental) that impact on risk assessment and treatment planning.<br>3. Describe basic pharmacology, principal interactions, including the implications of polypharmacy on oral health and overall health.<br>4. Perform safe transfer techniques and appropriate positioning aids where necessary.<br>5. Recognise and manage both verbal and non-verbal signs relating to pain, anxiety and phobia.<br>6. Provide preventive-focused dental treatment using behavioural, psychological and other non-pharmacological approaches in clinical practice.<br>7. Recognise the importance of prevention-led approaches in SND, including the use of Atraumatic Restorative Treatment	1. Manage medical emergencies, including palliative dental care, following local protocols and guidelines.<br>2. Integrate and evaluate medical, social, cultural, and environmental factors to develop comprehensive, patient-specific risk assessments and treatment plans.<br>3. Evaluate the implications for dental treatment and oral health care which arise from the effects of pharmacology and their impact on patient management.<br>4. Provide clinical care using behavioural, psychological and other non-pharmacological approaches.<br>5. Develop and justify individualised, risk-based dental care plans using clinical reasoning to provide evidence-based patient-centred care in diverse settings.<br>6. Apply mandatory reporting protocols in compliance with legal and ethical frameworks for abuse, demonstrating professional responsibility in safeguarding patients.

	(ART), Silver Fluoride, and other minimally invasive technique.<br>8. Recognise the signs of abuse and understand the legal and ethical responsibilities of mandatory reporting in the context of patient care.	
**Learning activities**	• Joint lectures with BDS and Medicine students on chemistry, physiology, pharmacology, and medicine.<br>• Use of "Delivering Better Oral Health" guidelines as a practical resource.<br>• Watching videos on medical emergency scenarios and learning appropriate responses.<br>• Pharmacology software exercises to understand drug interactions in patients with complex medical histories.<br>• **S**hadowing in SND clinic and domiciliary care for real-world patient interactions.<br>• Practice recognising verbal and non-verbal pain signs in patients.<br>• Demonstrations of medical and dental emergency management by St John Ambulance.<br>• Patient scenarios requiring students to interpret complex medical information, ensure patient safety, and understand the effects of medications and conditions on oral health.<br>• Reflective journals to document and analyse experiences and learning.	
**Assessment tasks**	• Case study discussions on various scenarios, including DBS selection, ART placement, and SDF application.<br>• Students are given a case scenario and must determine special needs-related considerations for treatment planning, requiring interpretation of medical information, risk assessments, and appropriate treatment planning, and identifying differences in treatment approach compared to a patient without disability.<br>• Demonstrating safe transfer techniques using positioning aids in the presence of a clinical assessor.	

8. Clinical Pathways and Team Approach to Care

Learning outcomes	Undergraduate	Postgraduate
	1. Explain the role of the carer and the importance of lived experience.<br>2. Explain the importance of the intra-interprofessional liaison in addressing patients' unique needs.<br>3. Demonstrate the ability to build interprofessional networks locally, providing guidance and group training to carers and other practitioners to support the delivery of appropriate oral healthcare.<br>4. Identify the value of teamwork, work collaboratively with dental specialties to advocate for relevant treatment options outside of individual scope of practice (knowing how, when and where to refer).<br>5. Practice responsibility for referring or arranging care.	1. Demonstrate understanding of the perspectives and lived experiences of patients and their carers or parents.<br>2. Adopt an interprofessional team approach to provide comprehensive, patient-centred care to optimise oral health outcomes.<br>3. Demonstrate leadership in care coordination, ensuring integrated and seamless patient management across healthcare providers.<br>4. Demonstrate understanding of the perspective of other service providers, managers of services and the voluntary sector.
**Learning activities**	• Attend interdisciplinary clinics and observe patient interactions across various disciplines.<br>• Participate in interprofessional education and collaborative research projects.<br>• Students present their own cases, discussing the management from an interprofessional approach.<br>• Role-play situations involving seeking support for complex treatment needs.<br>• Focus group discussions with carers, guided interviews on challenges in managing oral health care.	

	• Write a referral document detailing a patient's needs and rationale for referrals to other professionals.<br>• Students create an advocacy plan for both individual patients and larger groups, working in teams to design and implement the plan.<br>• Reflective essays on interprofessional collaboration experiences.
**Assessment tasks**	• Evaluate student's patient management plan and referral rationale through clinical assessment or oral presentation.<br>• Team dental students with pharmacy/nursing/medical students to create and assess a treatment plan for a hypothetical patient, considering overall healthcare provision.<br>• Team dental residents with public health graduates to create a program addressing the oral healthcare needs of special needs/geriatric populations, evaluating the understanding of clinical and public health barriers.<br>• Oral examination, where students propose strategies for interprofessional advocacy in a standardised patient scenario.

9. Dental Public Health and Oral Health Promotion

Learning outcomes	Undergraduate	Postgraduate
	1. Describe the principles of oral health promotion and common risk factor approach.<br>2. Explain the connection between health promotion and health policy development.<br>3. Demonstrate the capability to formulate individualised oral health education and prevention strategy for patients and their carers.<br>4. Describe the role of professional advocacy in promoting public policies, services and systems that respect diversity, equality and human rights.	1. Apply the theories and principles of oral health promotion to improve general health through oral health.<br>2. Develop, co-design and implement evidence-based oral health promotion strategies and programs in diverse community settings.<br>3. Apply social and environmental facilitators to enhance accessibility and effectiveness of oral health promotion programs.<br>4. Critically analyse and evaluate the effectiveness of current oral health policies and promotion campaigns for their inclusivity, effectiveness, and impact on different populations.
**Learning activities**	• Involvement in events supporting disability organisations to promote oral health.<br>• Field trip/community engagement programs related to public health promotion.<br>• Participation in oral health day, where students can meet with lawmakers and advocate for public health policies supporting vulnerable populations.<br>• Campaign design workshops, policy development simulation, and interdisciplinary seminars focused on oral health advocacy.<br>• Workshops with professionals in health promotion, focusing on interdisciplinary collaborations, e.g., kidney health and oral care.	
**Assessment tasks**	• Create an oral health promotional pamphlet or video targeting a specific group of patients with disability.	

	• Produce a poster for oral health using "Easy Read" pictures for better accessibility.<br>• Write a project activity report to evaluate the success and impact of a public health initiative.<br>• Develop individualised educational interventions for a patient with a disability, presented as a case study, class discussion, or written paper.<br>• Evaluate the success of a public health project and the student's ability to communicate this success to peers and supervisors.<br>• Postgraduate students could write a theory paper based on health promotion or conduct an evidence review (scoping or systematic review) on current oral health interventions and their impact on health promotion.<br>• Attend a governmental advocacy event and write a reflective essay about the experience, including thoughts on current legislation and its impact on health promotion for people with disability.

10. Clinical Research and Governance

<table>
<tr><th>Learning outcomes</th><th>Undergraduate</th><th>Postgraduate</th></tr>
<tr><td></td><td>1. Critically review relevant literature in order to practice evidence based special needs dentistry.
2. Demonstrate an understanding of scientific methodology and the ability to critically analyse published research and where appropriate, integrate this to the clinical situation.
3. Understand the importance of continuing professional development, lifelong learning, self-appraisal, peer review and competency in special needs dentistry.</td><td>1. Develop skills in undertaking and evaluating special needs dentistry and public health research.
2. Apply and interpret scientific methodology, critically appraising research for its clinical relevance and practical application.
3. Demonstrate an ability to critically evaluate all forms of information.
4. Apply contemporary research findings and scientific method in evidence-based dental practice.
5. Plan and evaluate one's own continuing professional development, lifelong learning, self-appraisal, peer review and competency.</td></tr>
<tr><td>Learning activities</td><td colspan="2">• Applying the Sackett model of Evidence-Based Dentistry.
• Conducting critical appraisals of peer-reviewed journal articles.
• Research proposal workshops, literature review assignments, and poster presentations.
• Group assignments involving literature searches to answer clinical questions.
• Lectures on biostatistics and training in search engines.
• Research seminars covering study design and methodology.
• Write reviews of current literature and identification of alternative care approaches.
• Recognising the use of Artificial Intelligence (AI) in research and guiding students on its best practices to evaluate evidence-based protocols.</td></tr>
</table>

	• Case studies to integrate research with clinical practice. Eg. A patient with Down syndrome who refuses treatment—students develop a communication strategy, outline diagnostics, and form a complete treatment plan.<br>• Developing clinical questions, conducting literature searches, applying critique tools, and synthesising information into decision-making.
**Assessment tasks**	• Research project assessed via a written thesis, presentations, and posters at university research groups/conferences.<br>• Systematic reviews, clinical audits, and capstone projects addressing patient care questions.<br>• Presentation of a completed research project for peer review and expert evaluation.<br>• Critical appraisal of research papers through written assignments or reflective journals.<br>• Journal article critique assignments, with students required to interpret and synthesise findings into clinical decision-making.<br>• Project-based work to assess EBD skills, including literature searches and application of critique tools.

References

1. Ford P, Farah C. Oral health therapists: what is their role in Australian health care? International Journal of Dental Hygiene. 2013;11(1):22-7. https://doi.org/10.1111/j.1601-5037.2012.00564.x

2. Bonvallet Commentz S, Mansilla Montenegro J, Bezanilla Fernandez F, Arancibia Flores I, Araya Salazar J, Astudillo Gutierrez D. Education's impact on primary care dentists' confidence in treating patients with special health care needs. Spec Care Dent. 2023;43(6):893-9. doi 10.1111/scd.12946

3. Chavez EM, Subar PE, Miles J, Wong A, Labarre EE, Glassman P. Perceptions of predoctoral dental education and practice patterns in special care dentistry. J Dent Educ. 2011;75(6):726-32. doi 10.1002/j.0022-0337.2011.75.6.tb05099.x

4. Koh D, Pradhan A, Aley M, Sohn W, Leadbeatter D. Oral Health Care for People with Disability: Curriculum Content in Oral Health Programs. Journal of Dental Education. 2024:e13894. https://doi.org/10.1002/jdd.13894

5. O'Rourke S, Dougall A, O'Sullivan M. Does education in special care dentistry increase people's confidence to manage the care of a more diverse population? Spec Care Dent. 2023;43(6):743-50. doi 10.1111/scd.12926

6. Australia Government, Disability Gateway. Roadmap – Australia's Disability Strategy 2021–2031. 2021.

7. Royal Australasian College of Dental Surgeons. Royal Australasian College of Dental Surgeons [Available from: https://racds.org/sdp/.

8. People with Disability Australia. PWDA Language Guide: A guide to language about disability. 2021.

9. World Health Organization. Framework for Action on Interprofessional Education & Collaborative Practice 2010.

10. Australian Dental Council. Professional Competencies of the Newly Qualified Dental Practitioner. 2023.

11. World Health Organization. Intersectoral Governance for Health in All Policies - Structures, Actions and Experiences. 2012.
